# Chemical Structure Comparison via Scanning Electron Microscopy of Spent Commercial Nickel–Metal Hydride Batteries

**DOI:** 10.3390/ma16175761

**Published:** 2023-08-23

**Authors:** Thomas Walther

**Affiliations:** Department of Electronic & Electrical Engineering, University of Sheffield, Mappin Building, Mappin Street, Sheffield S1 3JD, UK; t.walther@sheffield.ac.uk

**Keywords:** nickel–metal hydride (NiMH) batteries, ageing, scanning electron microscopy

## Abstract

Back-scattered electron imaging and X-ray elemental mapping were combined in a tabletop scanning electron microscope (SEM) to investigate cross-sections of three AA-type (mignon) nickel–metal hydride (NiMH) batteries from different manufacturers. All batteries underwent 500–800 charge/discharge cycles and reached their end of lifetime after several years as they could no longer hold any significant electric charge (less than 20% of nominal charge capacity), but none showed any short-circuiting. The types of degradation observed in this field study included electrode swelling, metallic nickel formation and carbon incorporation into pores in the positive electrodes and, in the negative electrodes, metal alloy segregation of different elements such as nickel, lanthanum and, in one case, sodium, as well as grain break-up and pore formation. All these phenomena could readily be observed at rather small magnifications. This will be important for the improvement of NiMH batteries, for which new generations with nominally slightly increased charge capacities are being marketed all the time.

## 1. Introduction

The quest for new electrode materials for use in rechargeable batteries is key to improving the charge capacity, lifetime and recyclability of such batteries, and the success of the whole approach of replacing fossil with renewable energy resources will depend on the future large-scale availability of efficient, safe and long-lived rechargeable batteries [1]. Nickel–metal hydride (NiMH) batteries are one specific type of secondary, i.e., re-chargeable, batteries, besides lead–acid, nickel–zinc, nickel–cadmium, sodium–sulfur, lithium ion or lithium-ion polymer batteries. The advantages of NiMH batteries include a moderately high specific energy density of the order of 100 Wh/kg at high specific power density [2,3], longevity, safety [4] and facile recyclability [5]. The disadvantages are the high cost for the metals nickel and cobalt, capacity reduction upon repeated cycling, and relatively high self-discharge [2]. NiMH batteries have been commonly used in electric vehicles [4] as well as small electronic goods. In this report, cylindrical AA-type (mignon-sized) 1.2 V batteries suitable for household appliances are studied.

The positive electrode is formed of nickel hydroxide (usually β-Ni^2+^(OH)_2_ [6] in the discharged state [7] as long as it remains Al-free [8,9]) that oxidizes to a mixed nickel oxide-hydroxide (Ni^3+^OOH) in the charged state by giving off a hydrogen ion during charging. This hydrogen ion is integrated into the negative metal alloy electrode during charging, turning it into a metal alloy hydride. Both reactions are reversible, and a discharged NiMH battery contains nickel hydroxide as the positive and a metal alloy as the negative electrode.

In order to suppress possible evolution of O_2_ at the positive electrode, small amounts of Co compounds, rare earth element hydroxides and/or Ca(BO_2_)_2_ are mixed into the Ni(OH)_2_ [10]. For the negative electrode various metal alloys are used, which can lead to quite complex crystallography and chemistry:

a. TiNi-based alloys form the cubic B2 or monoclinic B19′ structure [11];
b. AB_2_-based alloys (where A = Ti, V; B = Ni, Zr, sometimes also Cr, Co, Fe, Mn) form so-called Laves phases (cubic C15 [12,13], hexagonal C14 [12,14,15] or hexagonal C36 [16]);
c. AB_5_-based alloys are all hexagonal (where A = La, Ce, Nd, Pr; B = Ni, Co, Mn, Al) [17,18,19,20,21,22,23,24,25];
d. A_2_B_7_-based alloys (where A = La, Y; B = Ni, Mn) can be hexagonal 2H or rhombohedral 3R polytypes [24,26,27].

Only several studies seem to have studied the role of the strong caustic electrolytes, typically KOH or NaOH, used in NiMH batteries [20,28].

The main degradation mechanisms reported in the literature are the following:

  i. fragmentation, void formation and volume expansion (swelling) of the metal alloy of the negative electrodes [23,26,29,30];
 ii. surface oxidation of the metal alloy particles, especially AB_5_-type alloys [24,31] where La_2_O_3_ as well as La(OH)_3_ were observed. However, in addition to NiO, pure metallic Ni was also found [29,31]. This is in agreement with [20], who pointed out that, according to thermodynamics, the oxidation of La from LaNi_5_ cannot be avoided in the presence of KOH and AB_5_-type alloys will therefore oxidize selectively, where the less noble metals such as La, Ce, Nd, Pr, Mn or Al form an oxide scale and leave an enrichment in the more noble metals (such as Ni and Co) behind in the alloy surface layer. Dense oxide scales that are impenetrable to hydrogen could lead to gas leakage during overcharging and result in the premature dry-out of the cell [12];
iii. leaching of metals such as Zr and Mn [12] or Zn, Co and Mn [22] from the metal alloy into the separator, which could cause local short-circuiting.

The objective of this field study is to use analytical scanning electron microscopy to test which of these ageing mechanisms can be verified in commercial NiMH battery samples of different nominal charge capacity from different manufacturers. Additionally, we aim to establish whether any correlation can be made between the observed structure and chemistry of these samples and their specified charge capacity. By its very nature, such a field study of complete battery devices, rather than individual components thereof, must remain limited to small sample numbers and so each study can only be of limited statistical value. However, it is the only method capable of analyzing the root causes of long-term device degradation or failure under real-world operation conditions (rather than under artificial laboratory conditions). If several such studies were published by various groups worldwide, it would eventually also become possible to perform statistically meaningful meta-studies to compare device structures from different manufacturers, which may lead to future device improvements.

## 2. Materials and Methods

Commercial NiMH batteries from three different manufacturers that had undergone at least 500 charge/discharge cycles for 5–8 years during typical domestic use (that included partial and complete discharge, irregular re-charging—on the average once a week—and also light mechanical abuse in the form of occasional dropping onto floors) reached their end of life, holding 10–20% of their nominal charge. Two more batteries that had shown short-circuiting were excluded from this study. The remaining batteries listed in Table 1 were cross-sectioned in the laboratory via a process of cutting them into several 3 mm-thick discs with a diamond-coated brass blade saw under running water. After the electrolyte had leaked out, these discs were collected in a water bath and then ground from one side using SiC sandpaper with grit sizes ranging from P180 to P4000, corresponding to 6 µm particle size. The surfaces were carefully cleaned using 99.8% pure ethanol, acetone and lint-free paper, although this was performed without polishing with any type of suspension that could have been incorporated into the surface pores and falsified the analysis results. Hence, the only artefacts expected would be the occasional incorporation of SiC particles (which was not observed), some tissue fibres getting stuck in surface recesses (which could be removed with tweezers under an optical microscope) and the loss of small particles that had low surface binding and which could have been dissolved in or swept away with the solvents used.

The cross-sections were mounted via sticky carbon tape onto aluminum specimen holders that were inserted into a Hitachi (Hitachi High-Tech Europe, Krefeld, Germany) 3030+ tabletop SEM equipped with a secondary electron (SE) detector, a segmented solid-state detector for back-scattered electrons (BSE) and a silicon drift detector (SDD) for energy-dispersive X-ray (EDX) spectroscopy. The SEM was operated at a 15 kV acceleration voltage and 8.3 mm working distance. The width of the interaction volume for the X-ray generation of 15 kV electrons within matter was of the order of about 1 µm for GaAs and was similar for many transition metals. The X-ray detector was a 30 mm^2^ Bruker X-Flash430 SDD (Bruker Nano Analytics, Berlin, Germany) mounted at a take-off angle of 22° and equipped with an AP3.3 ultra-thin polymer window (MOXTEK, Inc., Orem, UT, USA) [32]. Bruker’s Quantax70 software was used to acquire and analyze spectrum images that store complete X-ray data at 10 eV spectral resolution for each of the 640 × 480 data points. From these, spectra were extracted and chemical maps were generated so that no prior assumptions about the presence of possible chemical elements had to be made. This is important as the metal alloys used can be very complex and no element must be overlooked. The acquisition times were around 1 h for each of the spectrum images. The pixel sizes ranged from 9 µm at 30× (Figures 4 and 6) to 2 µm at 120× (Figure 5). Unfortunately, no fresh, i.e., uncycled, batteries from the same batches were available, and a comparison with more recent batches could be misleading.

## 3. Results

**Figure 1:**
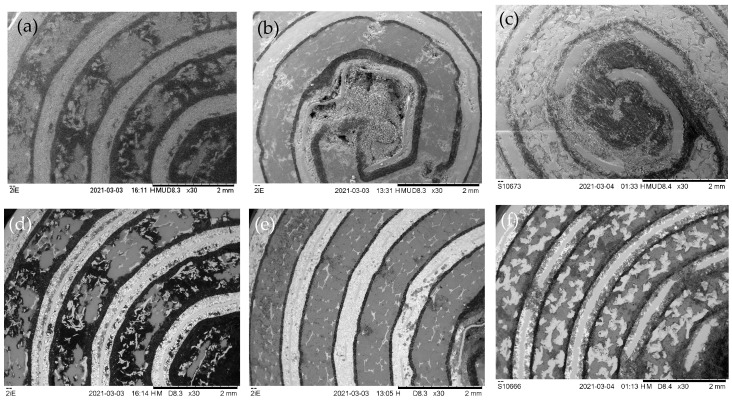
SE (**a**–**c**, top row) and BSE images (**d**–**f**, bottom row) of cross-sections of all three samples at low magnification of 30×. Horizontal field of view (hFoV): 5.6 mm. From left to right: (**a**,**d**) sample A, (**b**,**e**) sample B and (**c**,**f**) sample C.

SE images, as shown in the top row of Figure 1, are very sensitive to surface topology and reveal some large cavities near the central cores of all batteries where the rolled-up electrodes start. However, well-polished surfaces without large cavities are obtained elsewhere for samples B and C. Only the surface of sample A seems to contain numerous dents and cavities almost everywhere. All samples contain 4–5 windings of electrodes of comparable thicknesses so their total surface area at low magnification would be rather similar and cannot explain the large difference in nominal charge capacities. BSE images show stronger contrast due to changes in atomic numbers and so can be better used to distinguish different materials. The separators consist of polymers that mainly contain the light elements C, H and O and therefore appear dark in BSE imaging. Metals are generally much heavier and so appear brighter and allow us to identify the negative metal alloy electrodes in the discharged state. Oxides and oxyhydrides have an intermediate scattering power. As such, they appear in various interim greyscales and allow us to identify the positive electrodes (mainly Ni(OH)_2_ in the discharged state).

**Figure 2:**
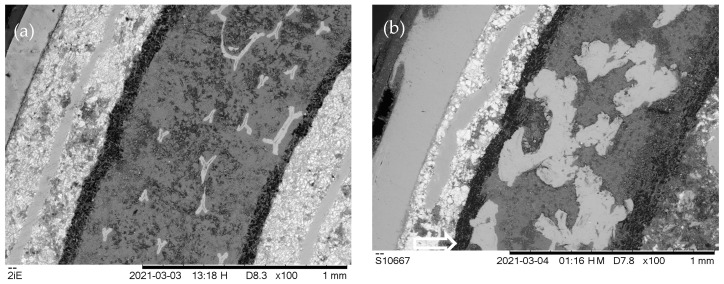
BSE images of cross-sections of outermost regions of cross-sections from samples B (**a**) and C (**b**) and at medium magnification of 100×. hFoV: 1.7 mm.

Figure 2 compares BSE images of outer parts of samples B and C at slightly higher magnification, showing two ≈0.2 mm-thin layers of polycrystalline metal powders that sandwich an approximately 0.5 mm-thick layer in the center that appears dark-grey, with light-grey embedded larger particles. The separators are the nearly black bands 0.05–0.1 mm-thin that run between the electrodes. The outermost layers at the top left are the cut metal cases of the battery housings.

Both negative electrode materials are extremely fine-grained, marked by <20 µm sample B in Figure 2a and <50 µm sample C in Figure 2b. In both cases, one can see clear signs of grain fracture as well as a separation into regions with predominantly bright contrast (heavily scattering elements) and darker contrast (less strongly scattering elements). Within the middle of the negative electrodes appear elongated, large, and smooth sections that, according to Figure 4 and from basic reflected light microscopy, can be identified as iron-based meshes within which the metal alloy particles were embedded and compressed into during manufacturing.

The wide region of the positive electrode in the center of Figure 2a shows differently shaped bright patches that are identified as cross-sectioned Ni foam (cf. Figure 4n) and many small dark spots that are presumably pits in the surface. The positive electrode of sample C in the center of Figure 2b shows much larger bright spots that are much more irregular in form also as bright as would be expected for pure Ni, in agreement with Figure 4o. These are far too large and irregular to be attributed to a possibly pre-existing Ni foam or Ni mesh and must have formed during repeated discharging and charging processes. Their wide lateral extension and frequent close proximity will form highly conductive nickel aggregates that extend throughout the positive electrode and, if penetrating deep enough into the separator, could eventually lead to local electrical short-circuiting. The onset of this can be seen in the large nickel grain in the bottom left of the positive electrode in Figure 2b, which has developed a finger-like extension (marked by a white arrow) that has grown ≈ 30 µm into the separator.

**Figure 3:**
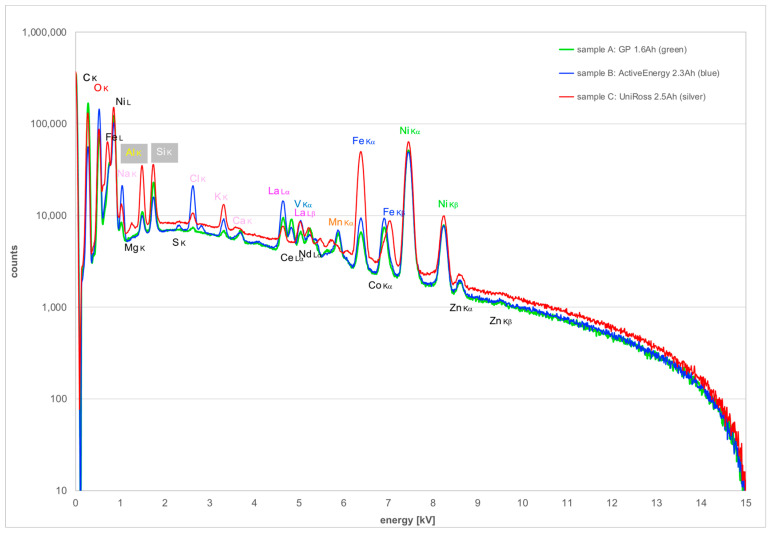
EDX spectra from the three sample areas shown in Figure 1d–f. Elements with labels above the peaks have been used for X-ray mapping in Figures 4 and 6, those with labels below are omitted because they showed only noise.

**Figure 4:**
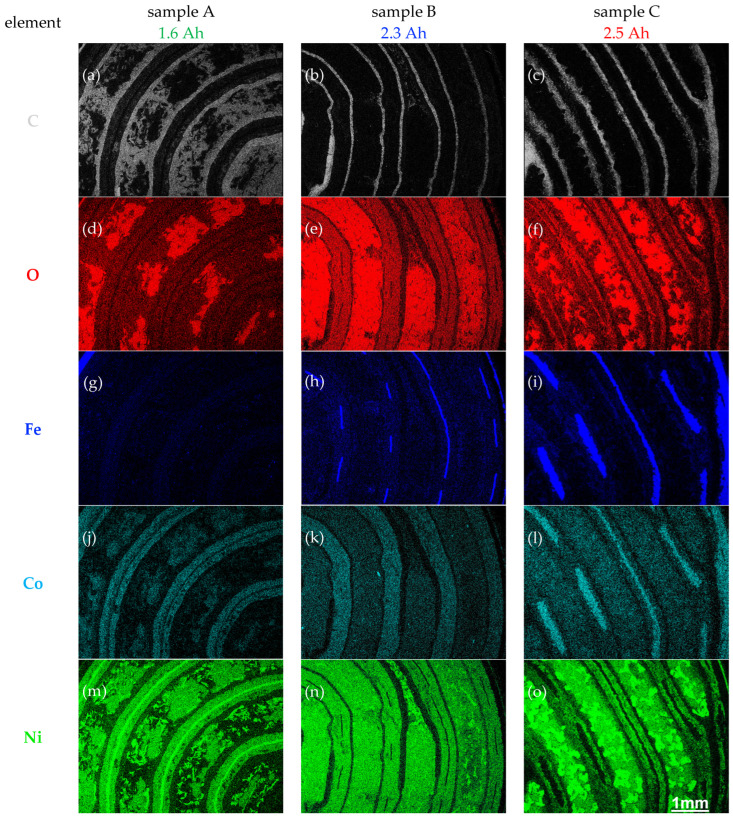
EDX maps of the major constituents from the same regions of each sample, including carbon, oxygen and the metals iron, cobalt and nickel. (**a**–**o**) Each element is shown in a different color in the rows; each column refers to a different sample. More maps from the same regions are shown in Figure 6. hFoV: 5.6 mm.

The integrated EDX spectra from regions shown in the lower row of Figure 1 are depicted in Figure 3. A logarithmic scale for the count rate has been employed to ensure that weaker X-ray lines are not overlooked. While there are no X-ray lines in the range of 10–15 keV energy, there is considerable overlap of X-ray lines in the 0–1 keV and 4.5–7.5 keV ranges, meaning that correct energy calibration as well as good energy resolution are necessary to identify lines reliably. The major X-ray lines are labelled above the corresponding peaks and have been used to generate elemental distribution maps, which are shown in Figures 4 and 6; weaker X-ray lines not used for mapping have been labelled below the peaks. There is a particular issue with the important element lanthanum (La) as this has several lines, all of which could overlap with other X-ray lines:

- The La Lα-line at 4645 eV potentially overlaps with Ti Kα at 4509 eV. However, because Ti L at 406 eV, as well as N K at 392 eV, are clearly absent even though O K at 525 eV is strongly visible, it can be concluded that there is **no** titanium in any of the samples and the La Lα-line can be used for mapping.
- The La Lβ-line at 5042 eV overlaps a little with V Kα at 4950 eV, but the 92 eV shift just suffices to allow the use of the latter line for vanadium distribution map generation.
- The La M-line at 836 eV overlaps strongly with Ni Lα at 853 eV, so none of these lines can be employed for mapping. However, La Lα- and Ni Kα can be used instead.

Figure 4 compares the X-ray maps from the elements C, O, Fe, Co and Ni of all three samples, leading to some important conclusions being drawn for each sample. In the following section, the three samples will be analyzed consecutively.

**Figure 5:**
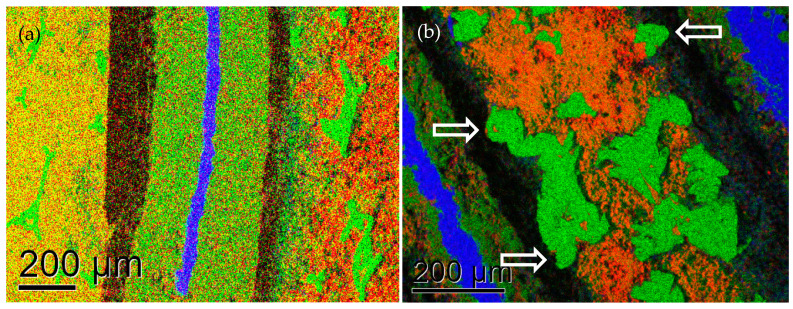
Representative composite EDX maps of cross-sections from samples B (**a**) and C (**b**) at higher magnification of 120×. hFopV: 1.4 mm. red: O, green: Ni, blue: Fe. Arrows indicate beginning dendrite growth into the separator.

**Figure 6:**
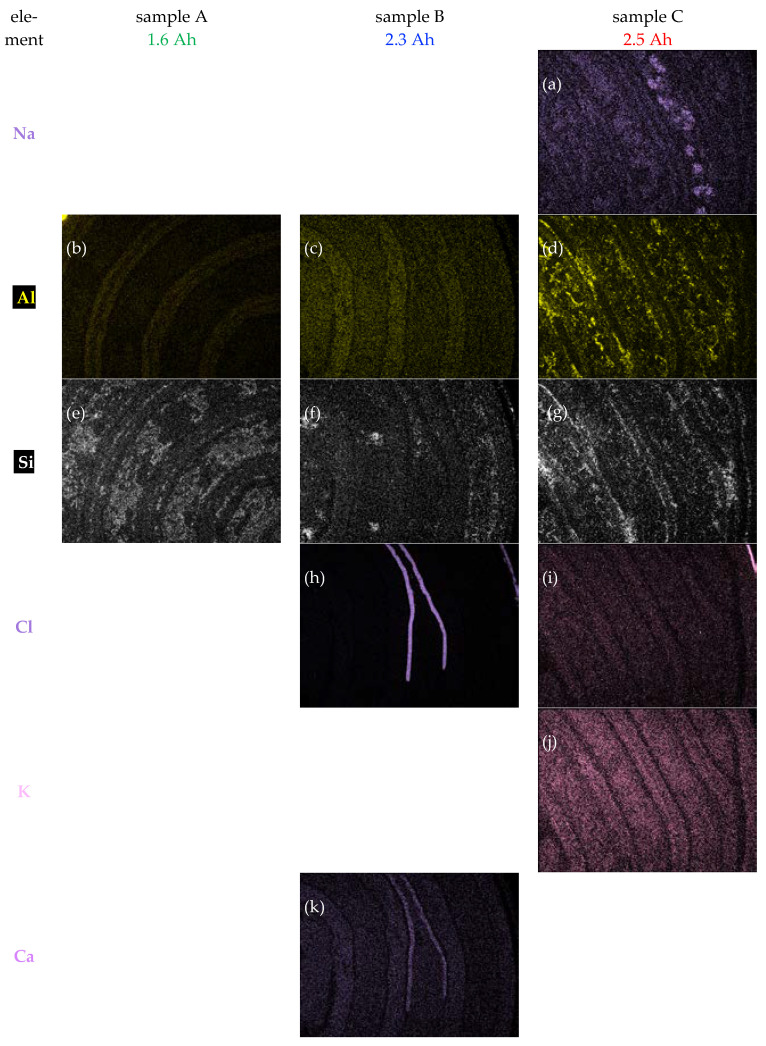

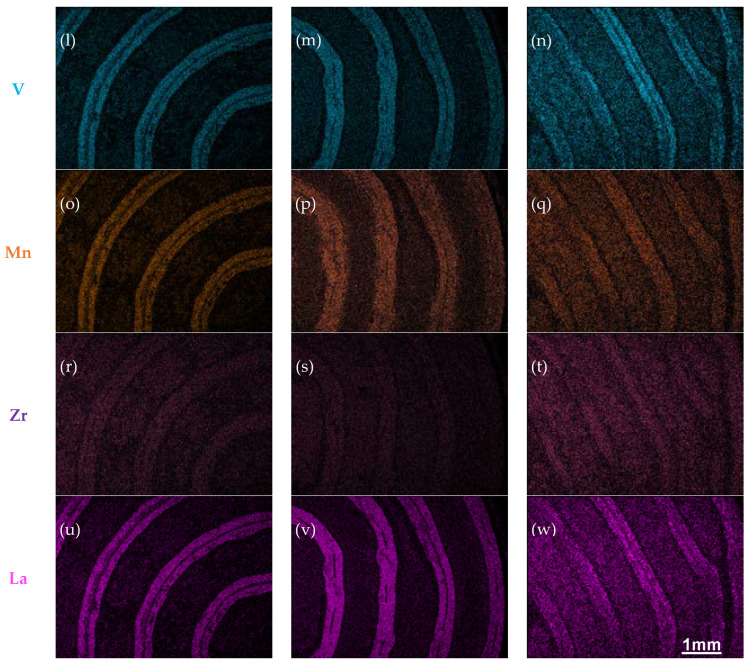
(**a**–**w**) EDX maps of less abundant elements from the same regions as shown in Figure 4. Maps that showed only noise, e.g., distribution of alkaline metals for samples A and B, have been omitted for clarity.

Sample A clearly has the thickest separator between the electrodes and also contains significant carbonaceous material between the sections within the positive electrode. The negative electrode contains Ni and Co (as well as Mn, Zr and La, according to Figure 6) and still appears to be rather compact, with only several darker parts in the BSE image correlating with corresponding intensity reductions in the elemental maps of Co, Ni, Mn, and La, which indicates some increase in porosity. The main deterioration, however, seems to relate to the disintegration of the positive electrode, which itself consists of some large and rather compact Ni(OH)_2_ particles separated by many much smaller, pure Ni particles embedded in carbon. Many of the latter Ni particles appear as Y-shaped platelets, which are also found in sample B and depicted in green in the left X-ray map of Figure 5a. These Y-shaped Ni particles are similar to those observed via Nomarski interference contrast in [30]. In summary, both electrodes show structural features in this sample that likely originate from repeated cell cycling and so occur due to ageing.

Sample B has a separator which is only 60–80 µm-thin, but there is also evidence for CaCl_2_ salt accumulation in some parts along it, as shown in Figure 6h,k. An iron mesh is embedded within the negative electrode, which itself consists of an alloy of Ni, Co, V, Mn, and La, but no Zr, and shows swelling in some areas. The positive electrode consists of Ni(OH)_2_ particles sintered around Y-shaped Ni particles that presumably stem from a nickel foam into which the NiOOH/Ni(OH)_2_ particles were intercalated during manufacture. The pure Ni particles are seen in Figure 5a as green, elongated features. The BSE image in Figure 2a shows that these Ni particles are 10–20 µm-thin, 70–250 µm-long and seem to consist of two parallel sections separated by a dark feature in their middle as if they had been formed by folding over a nickel foil after it had been cross-sectioned. The deterioration here seems to be due to swelling of the negative metal alloy electrode as well as some CaCl_2_ segregation between the separator and the positive electrode, which itself has remained largely intact.

Sample C exhibits a separator with large thickness fluctuations. A rather massive, 100–250 µm-thick iron–copper steel mesh is embedded within the negative electrode. This contains K, Ni, Co, V, Mn, Zr and La, wherein also small particles rich in Al and Si are visible. The positive electrode consists of a variety of small and large Ni particles as well as Na- and O-rich particles within a Ni(OH)_2_ matrix. Figure 4o and Figure 5b indicate the larger Ni particles partially penetrate into the separator (cf. white arrows in Figure 5b), suggesting that they grow during the charge–discharge cycles, in contrast to the pre-existing smaller Y-shaped Ni particles from the nickel foam used in the manufacture of the other two samples. The deterioration in this sample is linked to the formation of large Ni agglomerates protruding into the separator from the positive electrode, as well as Ni and La segregation within the negative metal alloy electrode.

Table 2 and Table 3 list the stoichiometry of all electrodes, excluding carbon and oxygen.

## 4. Discussion

None of the NiMH batteries investigated here contain noticeable amounts of Ti, Cr, Fe (other than in the casings and the metal meshes implanted into the negative electrodes, seen in Figure 4g–i), Sr or Y. The detection limit of SEM-EDXS depends on whether an element is enriched locally (then the detection limit would be lower overall as detection would be easier) or distributed equally (then it would be higher). It lies generally in the range of several at% in maps, as in Figure 4, Figure 5 and Figure 6, but can go down to typically 0.2–0.4 at% in spectroscopy mode, as in Figure 3. In the following section, the findings for positive electrode, separator and negative electrode will be discussed consecutively.

All positive electrodes contain Ni and O, as expected for Ni(OH)_2_. However, some Ni is clearly present as metallic nickel, either in the form of small Y-shaped sections of thin platelets made of nickel foam presumably used in the manufacturing (samples A, B) or large, irregularly formed Ni-rich grains formed during ageing (sample C). Beside Ni, sample A also shows some Si and Co within the Ni(OH)_2_ grains. Sample B shows Na and Co, sample C contains Fe, some rather evenly distributed Cl, K and Zr, larger lumps that are Na-rich, and many small precipitates on the electrode surfaces that are enriched in Al and Si. The composition of all elements found above the 1 at% level by EDXS (other than C, O) is shown in Table 2, where all elements are listed in order of decreasing concentrations. Here, all concentrations have been rounded to 1 at% = 0.01 and concentrations of elements below 0.005 have been omitted as they were close to the detection limits. The quantification did include all elements detected. The exceptions the light elements C and O, which cannot be reliably quantified due to detection efficiency, surface contamination and surface oxidation issues.

The origin of the formation of a dense stretch (likely a sheet in two dimensions) of CaCl_2_ between the positive electrode and the separator in sample B is not presently clear because, in the presence of KOH, this should have reacted to aqueous KCl and solid Ca(OH)_2_. This, however, was not observed. It is possible that, in this sample, some chloride salt was added to the electrolyte in order to inhibit corrosion and thus improve the cycle of life [33]. The separators in all samples seem to be still largely intact. However, particularly in sample C, some of the larger Ni crystallites formed extensions that penetrated up to halfway into the separator (as in Figure 5b), and these whiskers could have led to dangerous electrical short-circuiting in the future had this battery been subjected to further continuous use. As such, these developments would ultimately have limited the lifetime of such a battery.

All negative electrodes contain lots of Ni, although less than is contained the positive electrodes, as well as some amounts of Al (evenly distributed in samples A and B but strongly segregated in sample C), Co, La (very little in Sample C), Si (segregated towards the center of the electrodes in sample A but towards their surfaces in sample C), and Zr (very little in Sample B), so they are likely to be of the general AB_5_-type. As the spectrum images acquired contain full spectroscopic information at every data point, the average chemical composition of the metal alloys of the three different negative electrodes could be determined from different positions covering an area of about 1 mm^2^ in total, excluding any parts of the supporting Fe (sample B) or FeCo (Sample C) mesh, with the results listed in Table 3.

It can be seen that the alloys of the negative electrodes from samples A and B are rather typical AB_5_-type alloys (very Ni-rich, with significant additions of Co and La, and some Mn and Al), apart from some additional Na alloys only found in sample B. The alloy from sample C is completely different, however, with only about half as much Ni, high amounts of Si, Al and Fe, but hardly any Co or La. As Co and La are both rather expensive metals, this result means the materials needed for the alloy from sample C will have been much cheaper to purchase in comparison.

There is a weak O signal in samples A and B, where its even distribution along with the metals may just be due to the beginning surface oxidation that cannot be prevented after cross-sectioning in air; however, the O signal in sample C is much stronger and more indicative of surface corrosion of the negative electrode in the battery during service. This may be the result of the manufacturer’s choice to employ more cost-effective alloys (cf. above) and to include much more Al in the alloys of both electrodes. The correlation of Figure 4f and Figure 6d indicates that these factors have led to the formation of small alumina particulates.

The negative electrodes of the three samples have all shown distinct ageing characteristics. Sample A has developed internal pores within the negative electrode that have been partly filled by carbonaceous species, a procedure which presumably has only small electrical effects. The negative electrode of sample B has shown significant grain fracturing and swelling (Figure 2b), which could enhance significantly its internal resistance. The negative electrode of sample C has, due to its different chemistry (far less Ni; no Co or La, but instead much Si and Al), formed numerous silica and alumina particles (Figure 4f and Figure 6d,g) via internal segregation and corrosion that are expected to also increase the internal resistance to the vaccine, although they are not contiguous.

The main factors identified by Young and Yasuoka [34] as leading to a gradual charge capacity decrease in NiMH batteries, thereby limiting their lifespan, were γ-NiOOH phase formation and the subsequent particle break-up and/or increase in specific resistance in the positive electrode, as well as pulverisation and/or oxidation of the alloys making up the negative electrode. While direct detection of the γ-NiOOH phase would rely on diffraction techniques like electron back-scatter diffraction (EBSD) in SEM or transmission electron diffraction [35], neither of which have been employed here, electrode swelling, particle break-up and oxidation have all been observed in this study.

## 5. Conclusions

In summary, the various types of degradation observed include electrode swelling of the positive electrodes in samples A and C and of the negative electrodes in sample B; metallic nickel formation on the surfaces of the positive electrode in sample C; carbon incorporation into pores in the positive electrodes in sample A; weak segregation of different elements such as Na, Ni, V and La in sample C; formation of Al_2_O_3_ particulates in sample C; as well as grain fracturing and fine pore formation in all negative metal alloy electrodes, independent of their different chemistries.

The large Ni-rich grains that were formed mainly of sample C in the positive electrodes, as shown in Figure 2b and Figure 4o (and to a lesser extent, also in sample A), seem to emerge due to Ni segregation during charge–discharge cycling. This is the first direct evidence of pure nickel grains within the positive electrodes of aged NiMH batteries that the author is aware of. The reduction of Ni^2+^(OH)_2_ to Ni^0^ in the positive electrode seems to be correlated with the oxidation of metals in the complex alloys of the negative electrode which, again, is particularly pronounced in sample C. Which side of the redox reaction drives which is not presently clear, but both effects will mean that the formation of NiOOH in the positive electrodes and proton absorption in the negative electrodes during charging will be severely obstructed, thereby reducing electrical capacity.

If one wanted to use SEM as a real quality control tool, then it would be necessary to study the ageing of each battery type and batch individually by comparing at least one new and several aged batteries as a function of the electric charge they can still hold after being repeatedly fully charged. The problem here lies in the quick replacement of battery models by the manufacturers when they make minor changes to increase the nominal charge capacities so that each failure study conducted via destructive testing in an SEM can only provide a momentary snapshot for a given model and batch number that cannot be directly correlated with new batteries from subsequent batch numbers. To ensure future studies provide results on up-to-date battery types, models and batches, and accelerated ageing, it will be necessary to employ continuous charge–discharge cycles under controlled laboratory conditions rather, than in real-world domestic use, with typical weekly charging scenarios (under which 500 discharges would take 5 years). Both short-circuited aged batteries that were excluded from this study are to be investigated, and we will ensure in future studies that two unused batteries are always retained from each batch for reference, with one for use in electrical measurements (non-destructive) and one for cross-sectioning (destructive). This may help improve both the statistical significance and the general validity of our findings. As a destructive analysis technique, any electron microscopy of cross-sectioned batteries destroys the specimen so that no further electrical measurements are possible. As the electrolyte will inevitably be leaked out and lost (or contaminated if partially collected), only the electrode materials and their separator can be studied post-mortem. In situ or in operando electron microscopy has recently become available but can only study miniature sealed prototype cells, not real batteries.

A combination of X-ray mapping with EBSD is planned and may be useful to identify phases via their lattice types rather than by their chemistry. For example, for the positive electrode, a distinction between Ni(OH)_2_ and NiOOH based on elemental maps of Ni and O alone is impossible, and it is unclear whether the difference in BSE coefficients can be quantified reliably. For the negative electrode, more comprehensive X-ray spectroscopy maps and linescans could potentially reveal smaller changes as functions of ageing, such as the loss of specific minor elements by out-diffusion. The mapping of back-thinned regions in an SEM at a higher resolution may elucidate the issue more fully than was possible in this field study of potential metal segregation, examining materials such as that La shown here for samples B and C in Figure 6. However, the preparation of electron transparent thin foil sections is difficult with fine-grained, brittle materials such as NiOOH or the aged alloys used for the negative electrodes. Also, a field-emission SEM instrument will be needed for both EBSD and improved EDX mapping. Finally, given that many interesting irregularities here have been observed on the >10 µm scale, X-ray imaging via micro-computed tomography (µ-CT) of a whole battery may be used to identify regions of short-circuiting or preferential rare earth element accumulation in aged batteries. These batteries could then be located and prepared for the cross-sectioning of these specific parts for subsequent investigation using SEM-EDX mapping at higher spatial resolution. Scanning transmission electron microscopy (STEM) could even perform elemental mapping down at the nano-scale, but this will only make sense if the microstructure on the coarser µm scale has before been measured using SEM-EDX.

## Figures and Tables

**Table 1:** List of the three AA-type NiMH battery samples investigated.

	Sample A	Sample B	Sample C
Manufacturer	GP Batteries	Active Energy	UniRoss
Nom. charge capacity [Ah] when new	1.6	2.3	2.5
Main colour of casing	green	blue	silver & black

**Table 2:** Chemical composition of ***positive*** electrodes of battery samples from EDXS (excl. C,O).

Sample	Chemical Composition of Metallic Components
A	Ni_0.79_ Si _0.08_ Co_0.04_ Zn_0.03_ Fe_0.02_ Al_0.01_ Ca_0.01_ Na_0.01_
B	Ni_0.70_ Na_0.16_ Co_0.05_ Al_0.02_ Si_0.02_ Zn_0.02_ Fe_0.01_ K_0.01_
C	Ni_0.70_ Al _0.08_ Fe_0.07_ Si_0.06_ Na_0.04_ Co_0.02_ Zn_0.02_ K_0.01_

**Table 3:** Chemical composition of ***negative*** electrodes of battery samples from EDXS (excl. C,O).

Sample	Chemical Composition of Metallic Components
A	Ni_0.74_ Co_0.08_ La_0.05_ Mn_0.04_ Si_0.04_ Al_0.03_ Fe_0.01_
B	Ni_0.60_ Co_0.11_ La_0.10_ Na_0.06_ Mn_0.05_ Si_0.03_ Al_0.03_ Fe_0.01_ K_0.01_
C	Ni_0.33_ Si_0.23_ Al_0.19_ Fe_0.16_ K_0.02_ La_0.02_ Na_0.02_ Co_0.01_ Zn_0.01_

## Data Availability

The original data from the X-ray mapping (compressed .bcf file format which can be opened with Bruker’s Quantax or Esprit software) will be made available upon reasonable request to the author.
